# Factors affecting cytological results of endoscopic ultrasound guided-fine needle aspiration during learning

**DOI:** 10.1186/s13000-020-00938-8

**Published:** 2020-02-15

**Authors:** Jian-Han Lai, Hsiang-Hung Lin, Ching-Chung Lin

**Affiliations:** 1 Division of Gastroenterology, Department of Internal Medicine, Mackay Memorial Hospital, No. 92, Sec. 2, Chung-Shan North Road, Taipei, Taiwan; 2 Mackay Medicine, Nursing and Management College, Taipei, Taiwan; 3 Mackay Medical College, New Taipei, Taiwan

**Keywords:** Endoscopy, Fine-needle, Pancreatic neoplasms, Pancreatitis, Learning curve

## Abstract

**Background:**

Endoscopic ultrasound-guided fine needle aspiration (EUS-FNA) is a standard procedure used to obtain tissue samples for diagnosis of solid retroperitoneal tumours. However, this procedure demands high technical expertise and requires a strong learning curve. Our aim was to identify factors associated with false-negative EUS-FNA results during the learning for endoscopists.

**Methods:**

Our retrospective analysis was based on the EUS-FNA specimens collected by two novice endoscopists in 200 patients with retroperitoneal lesions who had confirmed image- or tissue-based diagnoses of malignancy or benign lesions.

**Results:**

In the first 40 performances endoscopists, the false-negative diagnostic rate of EUS-FNA was higher among patients with chronic pancreatitis than in patients without chronic pancreatitis. Patients who underwent FNA through the trans-duodenal puncture route also had lower success cytological diagnosis rate than through the trans-gastric puncture route. The rate of successful cytological diagnoses with EUS-FNA improved after 40 procedures and was not influenced by chronic pancreatitis presentation or difference puncture route.

**Conclusion:**

Regarding the learning curve, more than 40 procedures were required to achieve a stable success rate of EUS-FNA. Chronic pancreatitis and trans-duodenal puncture route are the predictive factors for a false-negative FNA cytological result during learning.

**Trial registration:**

This was a retrospective study.

## Introduction

Endoscopic ultrasound (EUS) is an important component of the assessment method for pancreatic cancer [1], with high resolution images of pancreatic lesions for diagnosis [2]. The use of EUS-guided fine needle aspiration (EUS-FNA) further extends the usefulness of EUS by providing cytopathological analysis and is less invasive than open procedure [3]. However, obtaining adequate specimens of solid pancreatic tumours by EUS-FNA is time consuming and technically challenging, requiring a learning curve for novice endoscopists [4]. Our aim in this study was to identify predictive factors for false-negative FNA cytological results for solid retroperitoneal tumours during the learning curve for two endoscopists who were novices on the EUS-FNA procedure, with the intent to improve the diagnostic accuracy for EUS-FNA.

## Materials and methods

Our study group included 226 patients who underwent EUS-FNA for diagnosing retroperitoneal solid lesions at a tertial hospital between May 2014 and March 2019. There were 3 patients with FNA technical failure in the study. There were 200 patients with a confirmed image- or tissue-based diagnosis of malignant or benign lesions. If a patient had an unsuccessful FNA cytological diagnosis, histological malignancy diagnosis was achieved by surgical biopsy. Those patients who were diagnosed with benign lesions underwent image follow-up for at least 6 months, for ruling out the possibility of missed diagnosis of malignancy. We excluded 23 patients in whom a final diagnosis was uncertain. Ultimately, our analysis was based on the data of 200 patients with a confirmed diagnosis, including 166 patients with malignancy and 34 patients with benign lesions.

The following personal and clinical data were extracted from the patient records for analysis: age, sex, chronic pancreatitis presentation, EUS findings (tumour location, tumour size and number of FNA passes), and cytological results. Adequacy of samples obtained was determined by the presence of well-defined pancreatic ductal epithelium or stroma cells of retroperitoneal mass [5], and the diagnostic criteria used for cytological diagnoses was determined by the cytopathologist. Negative means the ductal epithelium with a well-organized honey-combed pattern, uniform size nuclei, with fine granular chromatin and inconspicuous nucleoli; and malignant ductal epithelium which cells have lost the honey-combed arrangement, have varying nuclear size, irregularity in nuclear contour, vesicular nuclei and a prominent nucleoli. Based on the cytological results, 166 malignant patients were subdivided into the following 4 diagnostic groups: benign, atypical, suspicious, and positive [4, 6]. A “false-negative” FNA cytological diagnosis was defined as either a benign or atypical result, with a suspicious or positive finding of malignancy defined as a “positive” FNA diagnosis.

All EUS-FNA procedures were performed with patients in the left lateral decubitus position, under moderate conscious sedation using Midazolam 5 mg and Fentanyl 0.1 mg or Meperidine 50 mg. Additional sedatives were administered by the endoscopists, as needed.

As our study focused on the training period of EUS-FNA, all procedures were performed by two novice endoscopists who underwent an EUS and FNA training course. EUS procedures were performed using a curvilinear echoendoscope (GF-UCT260, Olympus, Japan), with aspiration performed using a 22-gauge needle (EZ Shot 2 or EZ Shot 3 plus aspiration needles, Olympus, Japan), which is standard in our institution. A fanning method was used for FNA, with aspiration from at least 4 different areas within the target lesion, with negative pressure applied using a 0–10 ml syringe. The aspirated tissues were collected and placed into a smear and cell-block preparation by the endoscopists themselves. Rapid on-site cytological evaluation was not performed. The endoscopists individually decided on the number of FNA passes required for each case based on the patient’s condition and volume of obtained tissue.

### Statistical analysis

Continuous variables were reported using their mean ± standard deviation, and categorical variables were reported as a frequency and percentage. Independent sample t-test, chi-square test and crosstabs statistics were used, according to the data type to compare baseline clinical characteristics between the positive and false-negative EUS-FNA diagnostic groups. Univariate analyses were used to identify predictive variables of a false-negative FNA cytological diagnosis. All analyses were performed using the SPSS 21.0 statistical package (SPSS, Chicago, IL, USA), with a two-sided *p*-value of 0.05 considered significant.

## Results

**Fig. 1:**
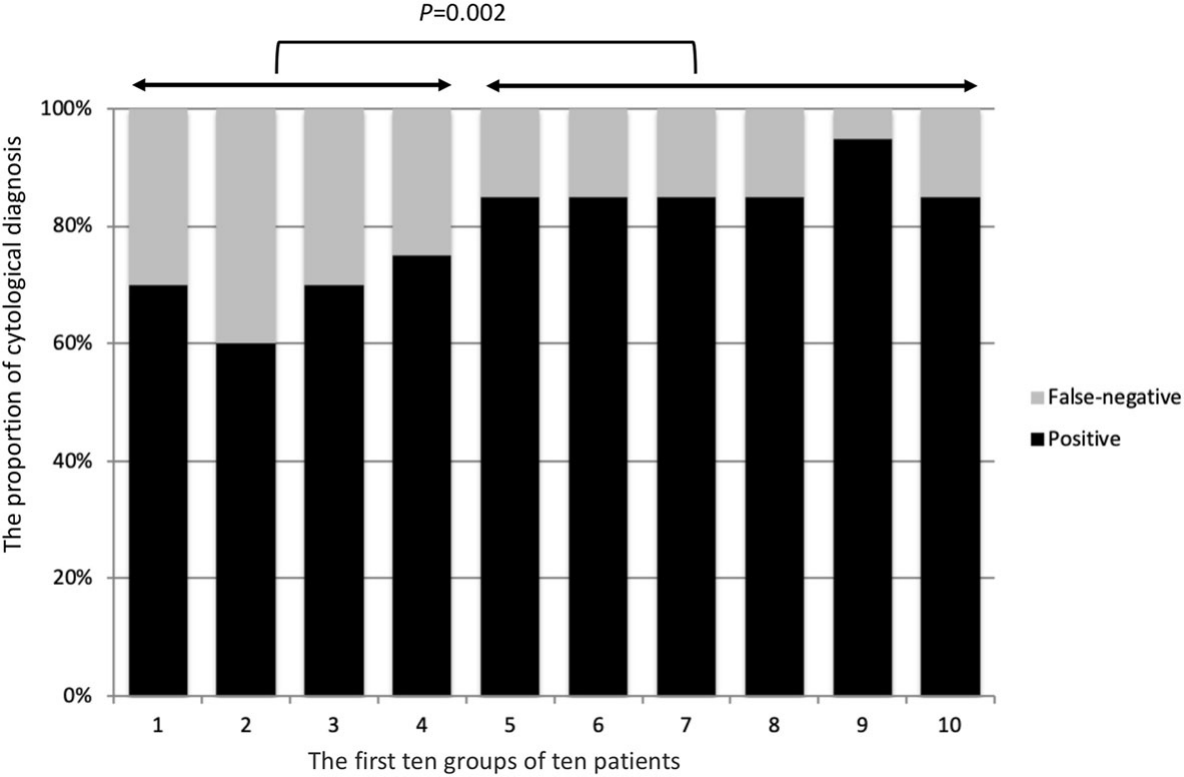
The proportion of positive and false-negative cytological diagnosis by endoscopic-guided fine needle aspiration in the first 10 groups of 10 patients

The FNA technical success rate was 98.7% (223/226). The 3 patients with failed FNA technique all had lesions of pancreatic uncinate process. The clinical and tumour characteristics, and the number of FNA passes of 200 patients are reported in Table 1. The mean age of our study group was 62.29 years (range, 22–98 years), and included 106 men and 94 women. The mean tumour size was 3.32 cm and the median number of FNA passes was 4 (range, 1–7 passes). The sample adequacy rate was 97% (194/200). The 6 patients whose samples obtained were inadequate had the diagnosis of adenocarcinoma, including 4 lesions at the pancreatic head. In total, 166 patients had final diagnosis of malignancy, including 136 adenocarcinomas, 19 neuroendocrine tumours, 8 lymphomas, 2 sarcomas, and 1 metastatic hepatocellular carcinoma. Two patients had mild pancreatitis after the FNA procedure, which improved after conservative treatment within 2 days. Cytological results are summarized in Table 2. Among the 166 malignant cases include in our analysis, a false-negative diagnosis was identified in 40 cases, 32 benign (false-negative) and 8 atypical. Among the 126 successful diagnostic cases, 92 were positive and 34 suspicious. The overall sensitivity, specificity, and accuracy of FNA cytological diagnosis were 75.9, 100 and 80%, respectively. The success rate stabilized to a level > 80% after about 40 procedures (Fig. 1). Furthermore, 3 procedures by the novice endoscopists with FNA technical failure occurred during the first 40 procedures which also included the 4 of 6 patients with inadequate tissue sample. Based on the above findings, we divided our data into two groups including “before (first forty)” and “after forty” groups. The success rate of FNA cytological diagnoses was significantly higher in the “after forty” group (86.7%) than in “first forty” group (65.8%) (*p* = 0.002). Furthermore, the number of FNA passes in the “after forty” group (3.83 + 1.17) was fewer than in “first forty” group (4.3 + 1.55).

**Table 1:** Clinical and EUS characteristics of patients who underwent endoscopic-guided fine needle aspiration (*n* = 200)

Age, years, mean ± SD (range)	62.3 + 14.3 (22–98)
Sex, male/female, n	106/94
Chronic pancreatitis, n (%)	37 (18.5%)
Tumor location, n^F1^	40/85/55/20
Tumor size, cm, mean ± SD (range)	3.3 ± 1.6 (0.3–12)
Numbers of FNA pass, median (range)	4 (1–7)
Adequate samples obtained, n (%)	194 (97%)
Malignant/ Benign lesion, n	166/34
Adverse events, n (%)	2 (1%)

*SD* Standard deviation

^F1^Pancreas uncinate process/head/ body/ tail/ others

**Table 2:** Fine needle aspiration cytological results for the 166 patients with confirmed malignancy

Cytologic results	n	
Benign (false-negative)	32	(27/3/2)^a^
Atypical	8	(7/1/0) ^a^
Suspicious of malignancy	34	(21/6/7) ^a^
Positive for malignancy	92	(81/9/2) ^a^

*NET* Neuroendocrine tumor

^a^Final histological diagnosis: adenocarcinoma/ neuroendocrine tumor, others

Personal and clinical characteristics were compared between patients in the positive and false-negative diagnostic groups (Table 3). Overall, the number of FNA passes, FNA puncture route, tumour size and location were similar in both groups. We further divided these patients into two groups of “first forty”and “after forty”, the chronic pancreatitis presentation, FNA puncture route and tumour size and location were also similar in both groups (Table 4). But the rate of successful cytological diagnosis in the “first forty” group was lower for patients with chronic pancreatitis (2/9 patients, 22.2%) than in patients without chronic pancreatitis (38/56 patients, 67.9%; *p* = 0.009), with an odds ratio of 5.6 due to unsuccessful diagnosis in chronic pancreatitis patient. For FNA conducted through the trans-duodenal puncture route, the successful cytological diagnosis rate (15/31 patients, 48.4%) was lower than that of FNA puncture through the trans-gastric route (25/34 patients, 73.5%) in the “first forty” group (*p* = 0.037). But in the “after forty” groups, there was no significant difference in the patients with chronic pancreatitis (*p* = 0.499) and FNA puncture route (*p* = 0.686) between successful and non-successful cytological diagnoses.

**Table 3:** Comparison of personal and clinical factors of 166 patients with malignancy between the positive and false-negative fine needle aspiration cytological diagnostic groups

Variable	Positive(*n* = 126)	False-negative(*n* = 40)	*P* value
CP, n (%)	13 (10.3%)	8 (20.0%)	0.114
Tumor location, n^F1^	16/42/32/13/23	8/17/7/5/3	0.063
Puncture route, n^F2^	57/69	23/17	0.086
Tumor size, cm	3.5 ± 1.5	3.2 ± 2.1	0.290
Pass number, n	4.2 ± 1.3	3.8 ± 1.6	0.093
Surgeon 1/2, n	67/59	15/25	0.085

*SD* Standard deviation, *CP* Chronic pancreatitis, *AP* Acute pancreatitis

^F1^Pancreas uncinate process/head/ body/tail/ others

^F2^ Transduodenal / Transgastric route

**Table 4:** Comparison of positive cytologic results with chronic pancreatitis and FNA puncture routes of 166 patients with malignancy during first and after forty experiences

	First forty (*n* = 65)			After forty (*n* = 101)		
	CP	No CP	*p*-value	CP	No CP	*p*-value
n, (%)	9 (13.8%)	56 (86.2%)		12 (11.9%)	89 (88.1%)	
PCR, n(%)	2 (22.2%)	38 (67.9%)	0.009	11 (91.7%)	75 (84.3%)	0.499
Pass number^a^	4.0 ± 1.5 (2–6)	4.4 ± 1.6 (1–7)	0.705	3.8 ± 1.5 (2–6)	3.9 + 1.2 (1–6)	0.400
OR (95% CI) of CP: 5.6 (1.26–24.84)						
Puncture route	Tranduodenal	Transgastric	*p*-value	Tranduodenal	Transgastric	*p*-value
n, (%)	31 (47.7%)	34 (52.3%)		49 (48.5%)	52 (51.5%)	
PCR, n (%)	15 (48.4%)	25 (73.5%)	0.037	41 (83.7%)	45 (86.5%)	0.069

*CP* Chronic pancreatitis, *PCR* Positive cytologic results, *n* Patient number

^a^Mean ± standard deviation (range)

## Discussion

This study presented an evaluation of the outcomes of EUS -guided sampling techniques based on the suggested guideline [7]. The false-negative results were further analysed to understand the clinical applications of the study. In our study, adequate specimen was obtained in 34 patients, but they had false-negative cytological results. Hence, we hope that these study findings could have implications in clinical work.

The sensitivity of EUS-FNA for the diagnosis of pancreatic tumour is good, with a rate of successful diagnosis of 74 to 97%. This result is similar to that reported in other hospitals that do not have rapid on-site evaluation, with a pooled diagnostic sensitivity of 89% [3]. Although our overall sensitivity of 75.9% is lower than this pooled result, it is in agreement with the sensitivity rate reported in a previous study during the learning of EUS-FNA (40–90%) [4]. The effect of various factors on the success rate of FNA diagnosis has been evaluated, including the size of the aspiration needle, comparison between the FNA technique and fine needle biopsy, negative pressure suction, and use of a stylet [8]. The cytological preparation method could also influence diagnostic accuracy [9]. In our study, the two endoscopists completed the same training course and had the same practice experience. Moreover, the EUS-FNA was performed using one common fanning and suction method, with the same cytological preparation used by both endoscopists. Cytological analyses were performed by cytopathologists who were blinded to the clinical diagnosis for all cases. Therefore, to specifically evaluate the effect of training, we controlled all possible factors, apart from patient demographic information, type of pancreatic disease, and tumour characteristics.

Our finding of a lower sensitivity of EUS-FNA in patients with chronic pancreatitis during first forty procedures, compared to those without chronic pancreatitis is noteworthy for clinical significance, and it is compatible with previous reports of a lower sensitivity in patients with chronic pancreatitis (53.5%), compared to patients without chronic pancreatitis (89.3%) [10]. The higher risk for a false-negative result in patients with chronic pancreatitis has been previously reported [11]. The diagnostic yield for patients with chronic pancreatitis may improve by increasing the number of passes [11, 12]. However, we did not identify an effect of the number of passes on the diagnostic success of EUS-FNA among patients with chronic pancreatitis. The rate of false-negative cytological results decreased after 40 procedures were performed without increasing the number of FNA passes or changing needle gauge or type. Hence, we attributed this to the experience level of the endoscopist in processing and deciding the smear while adjusting suction methods in next pass [12]. The level of experience of the endoscopist was also ascertained as a factor affecting the adequacy of samples obtained. Some studies attributed false-negative diagnoses to the technical difficulty in obtaining a sufficient tissue volume [13, 14], which means that obtaining sufficient experience is key to achieving a good diagnostic yield [15]. In our study, a stable diagnostic yield (> 80%) was achieved after performing > 40 procedures—a finding that was compatible with previously reported learning data [16–18].

As regards the FNA puncture routes, trans-duodenal lesions are more difficult compared with trans-gastric lesions, based on the puncture angle and relative anatomical complexities of the pancreas [19]. Varadarajulu suggested FNA should be performed with smaller calibre or flexible needle in trans-duodenal lesions [19]. In our study, the low rate of FNA cytological diagnosis was improved after 40 procedures without changing the FNA needles.

Our analysis of learning is, however, limited, as we included only 2 endoscopists, whose training centres and performance conditions were common. Moreover, we did not include procedure time or the effects of other EUS-related factors in our analysis of the diagnostic yield.

## Conclusions

We conclude that satisfactory EUS-FNA sampling may be achieved after a minimum of 40 procedures. In the first 40 procedures, patients with chronic pancreatitis or underwent trans-duodenal FNA route were at the risk of false-negative cytological diagnosis.

## Data Availability

yes

## Abbreviations

EUS: Endoscopic ultrasound
EUS-FNA: Endoscopic ultrasound-guided fine needle aspiration
FNA: Fine needle aspiration
